# Past, present, and future of Emergency General Surgery in the USA

**DOI:** 10.1002/ams2.327

**Published:** 2018-01-12

**Authors:** Heather G. Lyu, Peter Najjar, Joaquim M. Havens

**Affiliations:** ^1^ Department of Surgery Brigham and Women's Hospital Boston MA; ^2^ Division of Trauma, Burns and Surgical Critical Care Brigham and Women's Hospital Boston MA; ^3^ Center for Surgery and Public Health Brigham and Women's Hospital Boston MA

**Keywords:** emergency general surgery, quality improvement, surgical outcomes

## Abstract

Emergency General Surgery (EGS) patients represent a unique group of acutely ill surgical patients at high risk for death and complications. Since the inception of EGS as a surgical subspecialty in the early 2000s, there have been significant developments to further define the scope of EGS as well as to advance data collection, performance measurement, and quality improvement. This includes defining the EGS cohort by diagnosis and procedure and by overall burden, benchmarking of EGS outcomes, and creation of quality improvement programs aimed at reducing the excess morbidity and mortality associated with EGS. Going forward there exists a need for a more modern approach to quality improvement. This may include the creation of an EGS data registry, the use of electronic medical records data, wearable device technology, and a focus on patient reported outcomes.

## The Past – the birth of Emergency General Surgery

The earliest national initiative to standardize modern surgical trauma and emergent surgical care in the USA was born out of the military experience in the Korean and Vietnam wars. Injured soldiers were stabilized in forward surgical units and transferred to regional centers based on the acuity of their injuries. The effectiveness of this approach led to the creation of an accreditation system for trauma centers in the USA based on a hospitals' facilities and treatment capabilities.^1^ The American College of Surgeons Committee on Trauma (ACS‐COT) formed and pioneered regionalization of trauma centers in the 1950s. By 1987, ACS‐COT served as a verification program for trauma hospitals. This eventually led to the development of the Verification and Review Committee and Performance Improvement/Patient Safety programs to promote compliance to ACS guidelines for trauma and general surgery centers and to collect data on performance metrics.^2^ Trauma has become well recognized as a widespread public health problem and since the 1970s, it has become a focus of quality improvement, particularly with the creation and advancement of a comprehensive, standardized trauma registry, the National Trauma Data Bank (NTDB).^3^, ^4^ Many publications influencing interventions and guidelines based on NTDB investigations have been generated since its inception.^5^ However, non‐trauma surgical emergencies remained unrecognized as a public health concern at that time.

Emergency general surgery (EGS) was clearly defined, separate from trauma surgery and other general surgical specialties, for the first time in 2003 by the American Association for the Surgery of Trauma (AAST) and the American College of Surgeons Committee on Trauma. In August 2003, a summit meeting was held involving the leadership of the AAST, the ACS‐COT, the Western Trauma Association, and the Eastern Association of Trauma along with members from other relevant surgical societies. At this meeting, the field of emergency general surgery was clearly defined as a subset of acute care surgery, along with trauma and surgical critical care.^6^

In the USA, the incidence and prevalence of EGS conditions exceed that of other common, highly studied public health problems, including newly diagnosed cancers and new‐onset diabetes.^7^ More than 3 million patients are admitted annually to US hospitals with EGS conditions, representing over 7% of all US hospitalizations. Moreover, there are over 850,000 EGS operations performed annually in the USA.^7^, ^8^

The first non‐trauma emergency surgeries quality improvement programs included the Veterans Administration Surgical Quality Improvement Program, created by the Veterans Administration in 1991, as well as the ACS National Surgical Quality Improvement Program (NSQIP), which includes non‐VA hospitals and hospital systems.^9^ However, these programs are not specific to EGS as most of the data collected is from elective procedures and non‐operative cases are not captured. Emergency general surgery cases were defined and developed for the first time in 2013 by the AAST.^10^ The AAST published a landmark list of 621 International Classification of Diseases, 9th revision (ICD‐9) diagnosis codes that encompassed “any patient (inpatient or emergency department) requiring an emergency surgical evaluation (operative or non‐operative) for disease within the realm of general surgery as defined by the American Board of Surgery”.^10^ The published definition of EGS cases allowed for surgeons and researchers to study EGS as a field for the first time.

Since inception of EGS as a field in the early 2000s, there have been significant developments to further define the scope of EGS as well as to advance data collection and performance measurement.

## The Present

As EGS continues to mature as a surgical specialty, accounting for an estimated $28 billion in annual hospital costs in the USA, there has been a significant emphasis on data collection, quality, and performance measurement, and the development of evidence‐based guidelines and protocols.^11^ Since 2013, there have been further attempts to define the scope of emergency general surgery. A follow‐up study proposed 149 ICD‐9 procedure codes that could or would treat any of the previously defined 621 ICD‐9 diagnosis codes.^8^ In 2016, a study querying the National Inpatient Sample defined 7 procedure groups accounting for approximately 80% of the operative EGS burden throughout the USA.^12^ The findings allow for the development of EGS quality benchmarks that can be used to improve evidence‐based guidelines and create clinical decision support systems.

Recent studies have shown that EGS patients are at uniquely higher risk for medical errors and complications following surgery, with EGS patients up to eight times more likely to die compared to patients undergoing the same procedure electively.^13^, ^14^ Approximately half of all patients undergoing EGS will have a postoperative complication.^15^ In 2015, a study was carried out using the California State Inpatient Database that showed that 5.9% of EGS patients were readmitted within 30 days of discharge with higher rates of readmission in patients on public insurance and those with higher baseline comorbidity status, longer lengths of index hospital stay, and discharge dispositions other than home.^16^ Of those patients, nearly one in five were readmitted to a different hospital than where their surgery was performed, which was termed “care discontinuity”.^16^ In 2016, Medicare inpatient claims data were used to show that care discontinuity increased the odds of death in EGS patients by 16%.^11^ Patients treated at large, safety net hospitals and academic centers were more likely to have care discontinuity, highlighting a potential area for intervention.^11^

**Figure 1:**
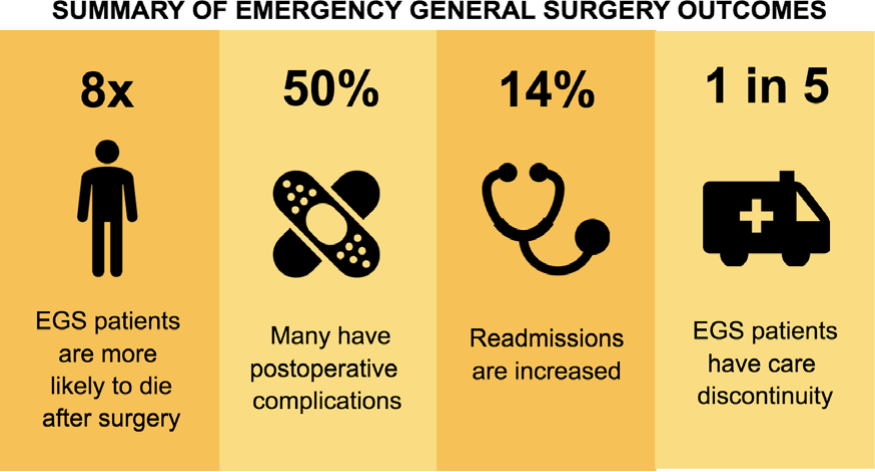
Summary of Emergency General Surgery (EGS) outcomes in the USA.

The EGS cohort is a unique, widely variable group of patients with high rates of death and complications (Fig. 1). The EGS outcomes research undertaken thus far has presented a multitude of opportunities for intervention and quality improvement. Nevertheless, there continues to be a need for focused quality improvement in EGS.

## The Future

Currently, most of the EGS outcomes research in the USA has been undertaken using administrative datasets, such as the National Inpatient Sample, as well as state inpatient databases. Although these databases capture many EGS cases, they do not include prospectively collected clinical data. The lack of clinical information in these databases limits the ability to appropriately risk‐adjust for the widely variable EGS patient population. An EGS registry modelled after the NSQIP and the NTDB was created in 2012 at Wake Forest University (Winston‐Salem, NC, USA) and is being studied by the ACS.^17^ The development of a robust national EGS registry will allow researchers and surgeons to undertake large, risk‐adjusted studies to create EGS‐specific benchmarks and risk stratification systems.

As technology advances, there are many opportunities to expand beyond the already‐envisioned national EGS dataset. Data from new sources such as wearable, mobile, and other device technologies are become increasingly relevant. For example, implementation of a telemedicine protocol using smartphones and digital cameras for assessment, treatment, and close follow‐up of minor pediatric burn wounds for patients at a local hospital in Poland with no specialized burn care had good results with easy access to burn consultations and multidisciplinary collaboration in the USA.^18^ Digital wearables can collect data about the human body before patients get sick, allowing physicians to personalize patient care.^19^ Nevertheless, while there is a dearth of mobile apps and new digital health technology, there is ample opportunity to create evidence‐based content with appropriate scientific and clinical support as evidenced by a recent review of apps for postoperative pain management.^20^

These data that were previously unable to be captured can now be used more readily as system capacities for data acquisition, storage, and processing are becoming more accessible and affordable.^21^, ^22^, ^23^ Electronic health records are allowing access to more granular data that can provide answers to clinical questions that were previously impossible to address. These large volumes of data can be translated to clinically relevant information as there are significant advancements in big data visualization and analysis techniques.^19^ Data stored in electronic health records can be utilized and visualized to track and report patterns of care and compliance to process measures and pathways. There is great potential in utilizing big data and health informatics. For example, a national EGS registry could be combined with machine learning analytics to create dynamic models to predict postoperative outcomes.

Patient‐reported outcome measures, standardized instruments designed to measure patient symptoms or quality of life measures, are being increasingly recognized as valuable information to guide patient care, improve population health outcomes, and increase cost efficiency.^24^ An emphasis on patient‐reported outcome measures is particularly important in a cohort proven to be at high risk and with less resources.^16^ Expansion of collection of health‐care data to include patient reported outcomes can assist in obtaining a better picture of the EGS patient group.

**Figure 2:**
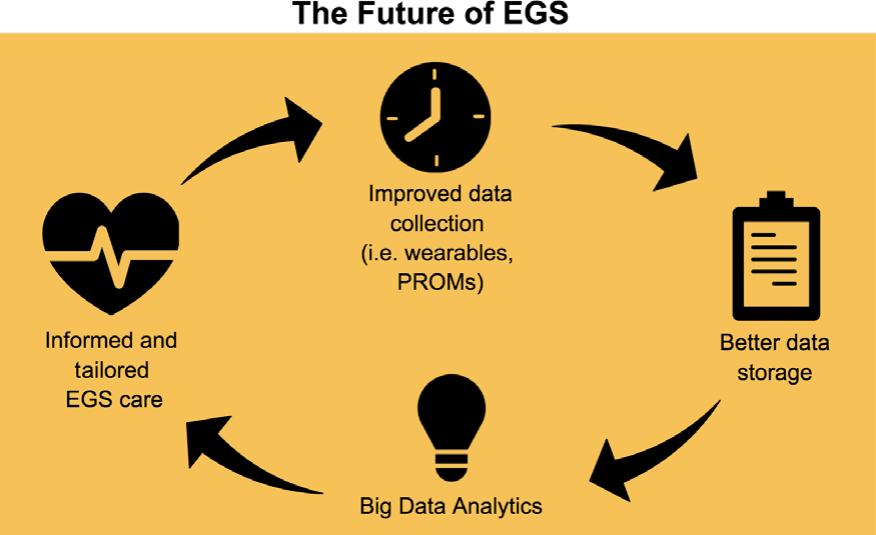
The future of Emergency General Surgery (EGS). PROMS, patient‐reported outcome measures.

Greater access to technology can provide EGS clinicians with more data than ever before. New data collection methods can be utilized to address the need for EGS‐specific process and outcome metrics as well as quality improvement programs (Fig. 2). Future improvements and developments in big data can inform and guide further growth of EGS as a new surgical specialty. The major challenges we face as we enter this era of new and ever‐evolving technology is to determine how we can best utilize this information to help us become better acute care surgeons rather than letting the technology drive how we care for our patients.

## Disclosures

Approval of the research protocol: N/A.

Informed consent: N/A.

Registration of the study: N/A.

Animal studies: N/A.

Conflict of interest: None declared.
